# Microcystin-Bound Protein Patterns in Different Cultures of *Microcystis aeruginosa* and Field Samples

**DOI:** 10.3390/toxins8100293

**Published:** 2016-10-12

**Authors:** Nian Wei, Lili Hu, Lirong Song, Nanqin Gan

**Affiliations:** 1 State Key Laboratory of Freshwater Ecology and Biotechnology, Institute of Hydrobiology, Chinese Academy of Sciences, Wuhan 430072, China; weinian710@163.com (N.W.); janicelili@126.com (L.H.); 2 University of Chinese Academy of Sciences, Beijing 100049, China

**Keywords:** microcystin, microcystin-bound protein, batch culture, semi-continuous culture, *Microcystis aeruginosa*, Lake Taihu

## Abstract

Micocystin (MC) exists in *Microcystis* cells in two different forms, free and protein-bound. We examined the dynamic change in extracellular free MCs, intracellular free MCs and protein-bound MCs in both batch cultures and semi-continuous cultures, using high performance liquid chromatography and Western blot. The results showed that the free MC per cell remained constant, while the quantity of protein-bound MCs increased with the growth of *Microcystis* cells in both kinds of culture. Significant changes in the dominant MC-bound proteins occurred in the late exponential growth phase of batch cultures, while the dominant MC-bound proteins in semi-continuous cultures remained the same. In field samples collected at different months in Lake Taihu, the dominant MC-bound proteins were shown to be similar, but the amount of protein-bound MC varied and correlated with the intracellular MC content. We identified MC-bound proteins by two-dimensional electrophoresis immunoblots and mass spectrometry. The 60 kDa chaperonin GroEL was a prominent MC-bound protein. Three essential glycolytic enzymes and ATP synthase alpha subunit were also major targets of MC-binding, which might contribute to sustained growth in semi-continuous culture. Our results indicate that protein-bound MC may be important for sustaining growth and adaptation of *Microcystis* sp.

## 1. Introduction

Cyanobacteria are photosynthetic prokaryotes commonly found in lakes, ponds, springs, wetlands and many other aquatic environments [1]. Rapidly growing cyanobacteria often form blooms that can have serious negative impacts on water quality and aquatic communities. Moreover, cyanobacteria can produce a large number of cyanotoxins, including microcystins (MCs), cylindrospermopins, anatoxins, saxitoxins and nodularins [2]. *Microcystis aeruginosa* is the primary producer of MCs. Over 90 MC analogues have been identified [3], among which microcystin-LR (MC-LR) is the most toxic and most commonly studied [4].

Microcystin is biosynthesized via mixed nonribosomal peptide synthetase/polyketide synthase (NRPS/PKS) pathways. Genes relating to the NRPS/PKS pathways constitute a large proportion of the cyanobacterial genome, and MCs are presumed to play an important physiological role [5]. Phylogenetic analyses have shown that the housekeeping genes (16S rRNA and *rpoC1*) and MC synthetase genes coevolved through the entire evolutionary history of MCs, indicating that MC synthetase genes are ancient [6]. Several studies have focused on the putative function of MCs in assisting toxic *Microcystis* cells to survive and dominate their environment. Schatz et al. [7] discovered that MCs released from cells could be sensed by the remaining *Microcystis* cells, prompting the remaining *Microcystis* cells to enhance MC production to cope with stress. External MC addition gained specific response from the *pksI-pksIII* gene cluster in *Microcystis aeruginosa* PCC7806 and its Δ*mcyB* mutant, further suggesting the role of MC as a molecular signal [8]. Moreover, MC at environmentally relevant concentrations (0.25–10 µg·L^−1^) enhances the size of *Microcystis* colonies, which may play an important role in the persistence of *Microcystis* colonies and the dominance of *Microcystis* in cyanobacterial bloom [9]. MCs are likely involved in enhancing photosynthetic adaptation of *Microcystis* to fluctuating inorganic carbon conditions [10]. MCs may also help to stabilize the photosynthetic apparatus against photo-oxidation and other oxidative stress in cyanobacteria [11,12]. In the early stage of iron stress, MCs may cooperate with the ferric uptake regulator FurA and downstream proteins to protect cells from damage by reactive oxygen species [13]. Metabolomic analyses of the response of an MC-producing strain and its MC-deficient mutant to high light exposure indicated that MCs play a pivotal role in high light adaptation [14]. Moreover, MCs could assist MC-producing strains in competing with non-MC-producing strains in nitrogen limited and ultraviolet B (UVB) radiation conditions [15,16].

In eukaryotes, MCs inhibit the activity of protein phosphatases 1 (PP1) and 2A (PP2A) by forming covalent bonds with their catalytic subunits [17,18]. The covalent bond is the product of a reaction between the unsaturated carbonyl of the methyl dehydroalanine residue of MC and the thiol of a cysteine residue of the protein phosphatase [18]. ATP-synthase beta subunit also has been demonstrated to be a target of MCs in eukaryotic cells [19].

By using differential fractionation of cell constituents, Jüttner and Lüthi discovered that in MC-producing *Microcystis* cells, the protein fraction that MCs bound to was primarily composed of phycobilins. The MC content was underestimated because protein-bound MCs did not bind to the C18 cartridges used for purification [20]. Vela et al. also found that MCs could bind to numerous proteins in vitro, including some unidentified proteins from a crude *Microcystis* extract [21]. In another study, Zilliges and colleagues [12] identified several specific proteins as MC-binding targets, including the essential Calvin cycle enzyme ribulose bisphosphate carboxylase/oxygenase (RuBisCO). MCs could form stable thioether bonds with cysteines of RuBisCO in the same way as observed in eukaryotic organisms. The binding was enhanced under oxidative stress, implying that MCs may modulate protein activities. Meissner et al. pointed out that numerous MCs that bound to proteins through conjugation were not detected by current MC analysis techniques including liquid chromatography-mass spectrometry (LC-MS) and enzyme-linked immunosorbent assay (ELISA) [22]. Collectively, these studies showed that MCs could bind to specific proteins, and that the level of protein-bound MC increased significantly under high light intensities and oxidative stress. However, correlations between the growth stage and the pattern and magnitude of protein-bound MCs remain unclear, thereby limiting the understanding of the role of protein-bound MC during growth.

In the present study, we investigated changes in the pattern and content of MCs (extracellular, intracellular free and intracellular protein-bound) during different stages of batch culture, as well as under semi-continuous culture and in field cyanobacterial blooms. We have identified several previously unreported MC-bound proteins in semi-continuous culture. Identification of the pattern of different MC fractions during culture growth and over field bloom seasons may clarify the role of MCs during growth of *Microcystis* and its adaptation to varying environmental conditions.

## 2. Results

### 2.1. MC Production and Release in Batch Cultures

**Figure 1:**
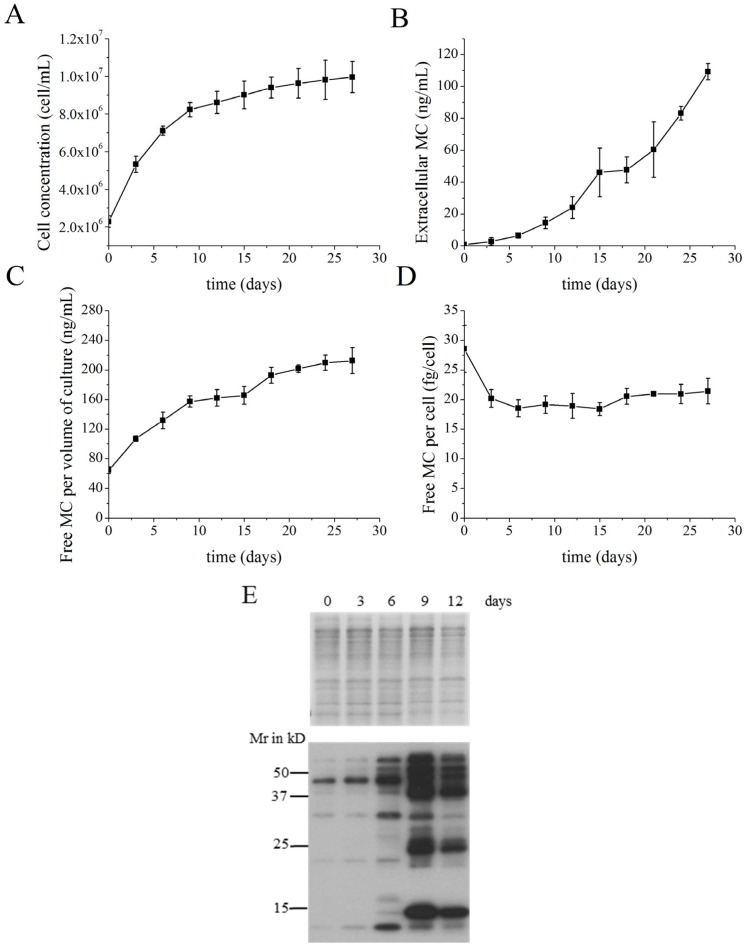
Changes in cell and microcystin (MC) concentrations in batch cultures of *Microcystis aeruginosa* FACHB-905. (**A**) Cell concentration; (**B**) Extracellular MC concentration; (**C**) Free MC intracellular concentration per volume of culture; (**D**) Free intracellular MC per cell; (**E**) Representative Coomassie-stained gel images (upper panel) and immunoblot analyses (lower panel) with anti-MC antibody detection of solubilized proteins obtained from cell debris after methanol extraction. The lanes were loaded with equal quantities of soluble proteins. Proteins were electrophoretically separated on 12% sodium dodecyl sulfate (SDS)-acrylamide gels. The samples were collected from cultures at day 0, 3, 6, 9 and 12. All values shown in panels **A**–**D** are means ± standard errors obtained from three replicates.

Batch culture experiments were designed to explore the production and release of MCs under conventional culture conditions. In the batch culture experiments, the cell concentration increased until the ninth day and then remained approximately constant with a slight increase until the end of the experiment at day 27 (Figure 1A). The extracellular MCs in the medium continued to increase during the entire growth period, from 0.75 ng·mL^−1^ to 109.24 ng·mL^−1^ (Figure 1B).

In the present study, free MC within inside cells was defined as MC that could be extracted by methanol and detected by high performance liquid chromatography (HPLC). Intracellular protein-bound MC was defined as MC retained in the debris, which contained the protein fraction after free MC removal. Protein-bound MC was determined by immunoblotting and detection with a specific anti-MC antibody. The free MC concentration per volume of culture (intracellular) reflected the corresponding growth curve, increasing in the exponential growth phase and remaining constant during the stationary phase (Figure 1C). The free MC per cell remained nearly constant throughout the growth period, which indicated that free MC concentration within cells did not vary with culture density (Figure 1D).

Coomassie-stained gel images show that an approximately equal amount of solubilized proteins from the MC-extracted residue was loaded onto each lane. The signal intensity of MCs bound to dominant proteins increased significantly as the cultures grew (Figure 1E). The protein-bound MC content reached its maximum when the cells were in the late exponential phase, at which point different MC-bound proteins were detected. 

### 2.2. MC Production and Release in Semi-Continuous Culture

**Figure 2:**
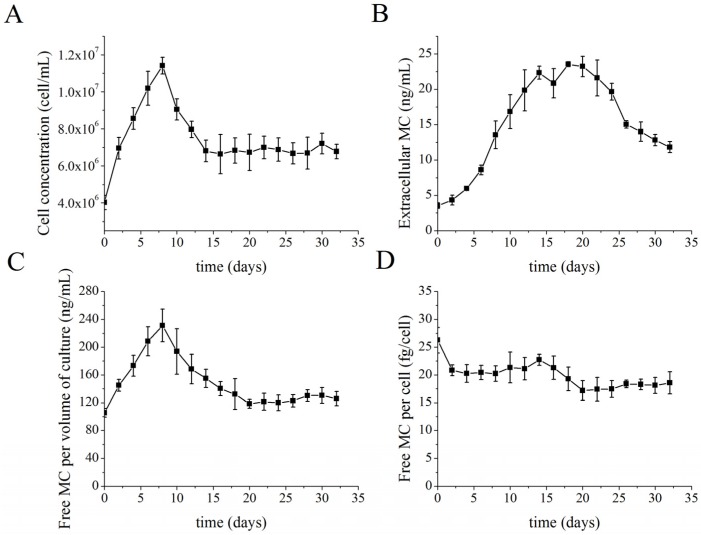

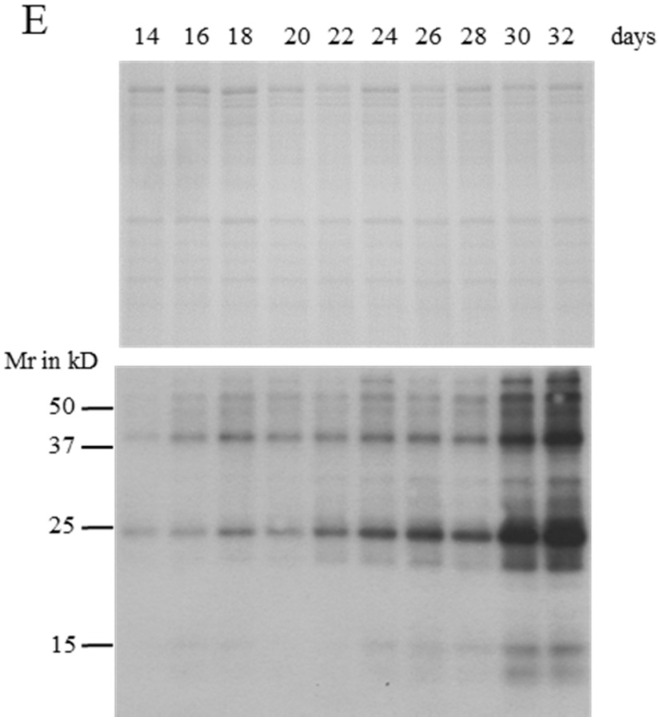
Changes in cell concentration and microcystin (MC) concentration in semi-continuous culture of *Microcystis aeruginosa* FACHB-905. (**A**) Cell concentration; (**B**) Extracellular MC concentration; (**C**) Free intracellular MC concentration per volume of culture; (**D**) Free intracellular MC per cell; (**E**) Representative Coomassie-stained gel images (upper panel) and immunoblot analyses (lower panel) with anti-MC antibody detection of solubilized proteins obtained from cell debris after methanol extraction. The lanes were loaded with equal quantities of soluble proteins. Proteins were electrophoretically separated on 12% SDS-acrylamide gels. The samples were collected from day 14 to day 32 with 2-day interval (in that time period cell density remained constant in spite of replacement of 25% cultures every two days). All values shown in panels **A**–**D** are means ± standard errors obtained from three replicates.

The semi-continuous mode was started on the eighth day of a batch culture. Twenty-five percent of the culture fluid was aseptically replaced with the same volume of fresh sterile BG11 medium every 2 days. From the start of the semi-continuous culture period, the cell concentration decreased for several days because the dilution rate exceeded the specific growth rate of the cultures (Figure 2A). When the specific growth rate matched the dilution rate, the cell concentration remained constant for a long period of time; this period was defined as the stable stage (days 14–32 in the present study).

The extracellular MC concentration increased in the early stage of the growth, reached a maximum concentration of 23.56 ng·mL^−1^ on the eighteenth day, and then decreased during the stage of stable cell density (Figure 2B). The trend in free intracellular MC concentration per volume of culture was similar to that of the corresponding growth curve (Figure 2C). The free MC per cell was stable before the time period when 25% culture replacement was begun (except on day 0 when it was at its highest concentration). A slight decrease was observed early in the stable stage of culture density (Figure 2D).

In semi-continuous cultures, the dominant MC-bound proteins were the same during the entire time period (Figure 2E). However, the quantity of protein-bound MCs increased gradually during the early stable stage, and dramatically at the end of the sampling period.

### 2.3. MC Production in Field Samples from Lake Taihu

**Figure 3:**
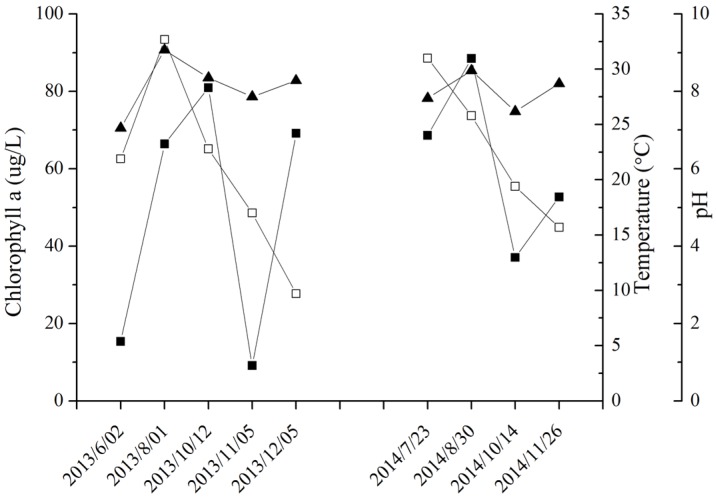
Environmental parameters in Meiliang Bay, N2, of Lake Taihu, from 2013 to 2014, chlorophyll a (■), temperature (□) and pH (▲).

As shown in the Figure 3, chlorophyll a concentration was highest in October 2013 and August 2014. Temperature was highest in August 2013, and decreased gradually from August to December. A similar temperature profile occurred in 2014. The water pH ranged from 7.05 (June 2013) to 9.07 (August 2013). The content of free MCs per unit dry weight during the algal bloom seasons was highest in October (1063.02 μg·g^−1^) and November (1169.50 μg·g^−1^) 2013, and in July (1030.28 μg·g^−1^) and October (1500.67 μg·g^−1^) 2014.

**Figure 4:**
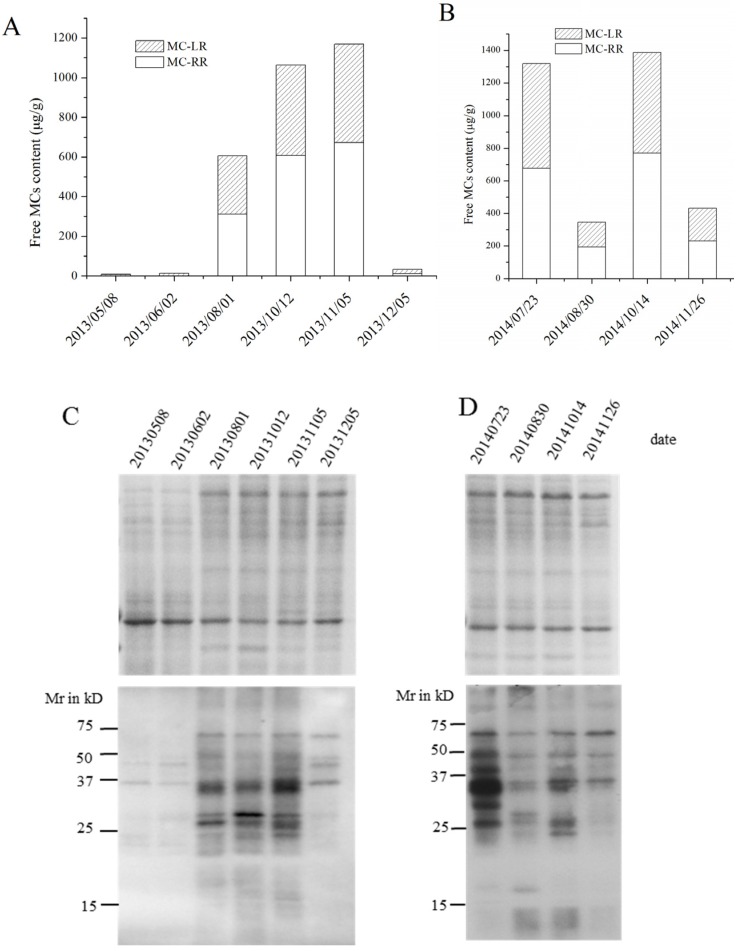
Changes in microcystin (MC) concentrations in field samples taken from Taihu Lake during 2013 and 2014. (**A**) Free MC content per dry weight in samples from 2013; (**B**) Free MC content per dry weight in samples from 2014; (**C**) A representative Coomassie-stained gel image (upper panel) and immunoblot analyses (lower panel) of solubilized proteins obtained from cell debris after methanol extraction of samples from 2013, using an anti-MC antibody; (**D**) A representative Coomassie-stained gel images (upper panel) and immunoblot analyses (lower panel) of solubilized proteins obtained from cell debris after methanol extraction of samples from 2014, using an anti-MC antibody. The lanes were loaded with equal quantities of soluble proteins in both **C** and **D**. Proteins were electrophoretically separated on 12% SDS-acrylamide gels.

SDS-polyacrylamide gel electrophoresis (SDS-PAGE) and Western blotting results from the field samples from Lake Taihu during the algal bloom seasons of 2013 and 2014 are shown in Figure 4C,D. The protein banding patterns seen in the SDS-PAGE gels were not significantly different between samples. The amount of MC bound to specific proteins in 2013 was higher in August, October and November than May, June and December. In 2014, the protein-bound MCs were much more abundant in July and October than in August and December.

### 2.4. Identification of MC-Bound Proteins

**Figure 5:**
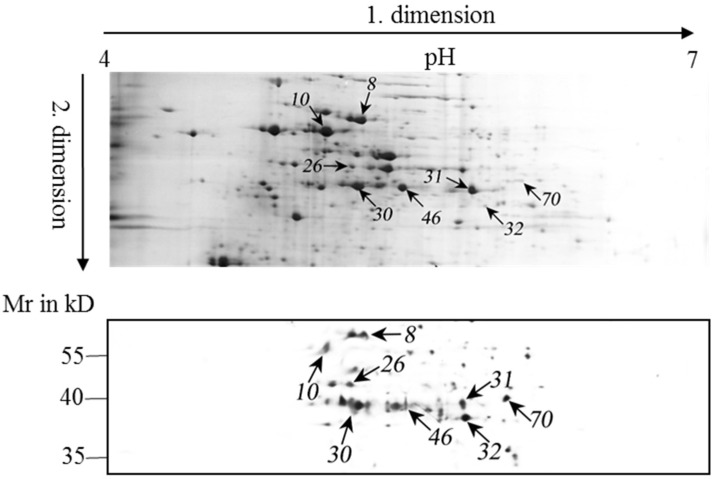
Representative Coomassie Blue-stained two-dimensional electrophoresis gel images (upper panel) and immunoblot analyses (lower panel) using an anti-MC antibody. Proteins were solubilized from the debris that remained after methanol extraction of cells that had been grown in semi-continuous culture. The numbered arrows indicate spots in the images that were selected for mass spectrometric analysis.

Proteins that bound to MC during semi-continuous growth were further analyzed. Eight spots on 2-D gels which appeared prominently after MC-antibody detection were chosen for mass spectrometric analysis (Figure 5). Various characteristics of these proteins are listed in Table 1. All of these proteins have at least two cysteine residues. The identified proteins were classified according to their biological function as provided in the Uniprot database and functional annotation by Blast2GO (BioBam, Valencia, Spain).

## 3. Discussion

The production of MCs is frequently reported by gravimetric (MCs per dry weight), culture volumetric (MCs per volume of culture) and cell quota (MCs per cell) determinations. As cells accumulate carbohydrates in the latter growth phase [23], there is an increase in the cell density which thereby causes misleading results in measurements related to weight. We therefore chose to use the culture volumetric concentration and cell quota to estimate MC production in this study. The changes in free MC concentration per volume of culture corresponded with growth curves. This was similar to the results of Orr and Jones [24], indicating a correlation between growth and free MC production. The free MC per cell of the batch culture and the semi-continuous culture were almost constant during the exponential growth phase, except for significantly higher levels on day 0 (Figure 1D and Figure 2D). The higher intracellular free MC concentration on day 0 may be attributable to the high cell concentration of the parent culture used for inoculation, since previous research has reported that high cell abundance can result in a marked increase in MC quota [25]. In semi-continuous cultures, the slight increase and then significant decrease of cell free MC concentration during the adaptation stage and in the early stable stage, respectively, were deemed to be reflections of MC production when cells encountered a drastically changed environment. Wood et al*.* [25] demonstrated that cell abundance and mutually correlated environmental parameters could affect MC synthesis; i.e., the dilution of the culture may bring about variable production of MCs. The constant cell-free MC concentration in the later stable stage of growth demonstrated balanced production and metabolism of free MCs in cells, as also shown by the constant level observed in batch cultures. These results are in accordance with the studies of Wiedner and colleagues [26]. They also suggested that MC production was constitutive in *Microcystis* strain and that MC-producing strains established a stable cellular MC content within a certain range. The free MC concentration within cells may has particular meaning for *Microcystis* cells, considering the multiple proposed functions of MC.

Changes in the profiles of proteins containing bound MCs in batch and semi-continuous cultures are presented in Figure 1E and Figure 2E. In batch cultures, when reaching a specific cell concentration or late exponential growth phase, new MC-bound proteins emerged and replaced the former dominant MC-bound proteins. However, when the cell concentration was kept relatively constant and the culture was stabilized in exponential phase at stable stage, as shown in semi-continuous cultures, no such replacement occurred. Seemingly, MCs become bound to different proteins as an adaptive advantage to stress, since in the later period of batch cultures, cells were subjected to nitrate limitation, phosphate limitation, low dissolved CO_2_ content, high pH, light limitation due to self-shading and oxidative stress [27]. Cells in semi-continuous culture did not experience these stress conditions. Since protein-bound MCs may play roles in maintaining protein function [12] and the different proteins that MCs bind to probably participate in different physiological functions, protein-bound MCs may regulate cell metabolism and modulate the activities of specific proteins. In the early exponential phase, the gradual increase of MCs bound to specific proteins indicated that greater amounts of MC-bound proteins participated in physiological processes during the rapid growth of cells, while proteins related to growth and stress response were co-expressed under stress conditions, as shown in the protein band from the ninth day of the batch culture in Figure 1E. Later on, more MCs bound to proteins related to stress response, rather than to the proteins to which MC bound earlier in the growth phase. In the semi-continuous culture, more MCs bound to specific proteins were needed to sustain rapid growth. The different MC-bound protein pattern and free MC tendency between batch and semi-continuous cultures may reflect possible regulation of MC during growth. Considering these various changes and their correlations, we suggest that protein-bound MC may be important for sustained growth.

Rohrlack et al. [28] studied the fate of radioactively-labeled MCs in cells and discovered that MCs were not subjected to export or intracellular breakdown under high or low light. Data obtained from field samples indicated that release of MCs from cells occurred during the senescence and decomposition period of *Microcystis* cells [29]. According to that observation, the gradual increase of extracellular MCs in the present study could be attributed primarily to cell aging and lysis, which are typical during the stationary phase of batch cultures. Nevertheless, considering the existence of a putative ABC transporter gene, *mcyH*, in the MC synthetase gene cluster [30], the possibility that MC may be exported from *Microcystis* cells by McyH cannot be ignored. The decrease in extracellular MCs in the stable stage of semi-continuous cultures may be correlated with the halted excretion of MC. The newly synthesized MC may instead selectively bind to proteins, as indicated in Figure 2E.

Field samples were analyzed in order to investigate the profile of MC-bound proteins and total MC during succession of natural cyanobacterial blooms. Lake Taihu was chosen for its annually recurring dense *Microcystis* blooms. Hu et al. [31] sampled the same site from 2012 to 2014. They determined that the MC content per unit dry weight correlated with the percentage of toxic *Microcystis*. Thus, higher free MC content is attributed to more toxic *Microcystis* in a sample. As shown in Figure 4C, the amount of MCs bound to proteins was higher during bloom-sustaining periods, and decreased by December when the bloom began to collapse. In the July 2014 sample, the bloom outbreak month, more MCs bound to specific proteins. It was also found that in samples with higher free MC content per dry biomass, MC binding to specific proteins was also relatively high. This correlation has also been found in the samples from German lakes [22]. The chlorophyll a concentration in Lake Taihu exceeded 10 µg·L^−1^ from July to October during both sampling years (Figure 3). The chlorophyll a concentration (representing the phytoplankton biomass), temperature, light and pH were all highest during the bloom sustaining period (Figure 3, Figure S1). The results are consistent with the observation of Su et al [32]. Many studies have shown that higher elevated temperatures and pH values favor the production of MCs [32,33,34]. The higher free MCs and protein-bound MCs in samples during summer and early autumn may relate to these physiochemical parameters.

During the summer and early autumn, phytoplankton cells experience harsh conditions such as high light intensity, high temperature and nutrient limitations, which are unfavorable for their growth. The observation that *Microcystis* cells can sustain their growth and form blooms under these conditions is generally attributed to their physiological advantages, including colony formation, buoyancy regulation and efficient utilization of nutrients [35,36,37]. The results of this study indicate that protein-bound MC may also participate in the adaptation of *Microcystis* cells to stress environments, as evidenced by the finding that in Lake Taihu the amount of protein-bound MC was higher during the summer and early autumn. Zilliges et al. [12] found that the quantity of protein-bound MC increased significantly under high light intensity and oxidative stress, and that MC binding to a specific protein could sustain chemical conformation of the protein and maintain its bioactivity. These results indicate that protein-bound MC may be beneficial to *Microcystis* cells in environmental adaptation and dominance.

The results of Meissner et al. [22] and results reported here indicate that protein-bound MCs make up a considerable proportion of the total MC in field samples. Meissner et al. considered that the protein-bound fraction of MC may not need to be included in total toxicity assessment, due to the seemingly irreversibility of MC conjugation. The view is recently challenged in research by Miles and colleagues [38], which discovered that conjugation of MCs with thiols is reversible, with higher pH promoting deconjugation. They also suggested that certain other conditions, including temperature, free/protein-bound MC concentration and pKa values of the thiols present could influence the equilibrium between conjugation and deconjugation. The reversibility could be possible, as the conjugation reaction between an α,β-unsaturated carbonyl of MC and free thiol of the cysteine of specific biomolecules/proteins is a Michael-type addition reaction [39] and several studies have also discovered that some Michael additions of oxygen, nitrogen and sulfur nucleophiles to α,β-unsaturated carbonyls were reversible [40,41,42,43]. Miles et al. [38] pointed out that the environmental and toxicological consequences of the reversibility in aquatic organisms need further investigation. A similar view was also presented by Schmidt et al. [44] who proposed that more studies on this reversible conjugation are essential for complete understanding of the toxicity, transport and transformation of MC in living cells. With respect to reversibility as well as the high amount and the exposure of protein-bound MC, we suggest that the fraction of protein-bound MC should not be ignored and should be taken into account for risk assessment of MC. For instance, the protein-bound MC fraction may be quantified and calculated into total MC content when assessing the toxicity of field algal samples and aquatic products. 

Although the phytoplankton community in Lake Taihu differed from one month to another, the profile of MC-bound-protein bands was similar among samples from different months in both 2013 and 2014. This may partially be due to the large proportion of *Microcystis* in the detected samples (ranging from 40% to 80% of the total biomass, data unpublished). *Microcystis* was the only MC-producing genus of cyanobacteria in Meiliang bay of Lake Taihu [45]. A combined influence of various factors (biotic and abiotic) may determine the specific MC-bound proteins in samples from Lake Taihu. This phenomenon need to be further investigated. The dominant MC-bound protein bands differed in laboratory and field samples. This may be explained by the observation that field samples often include a variety of diverse phytoplankton, a quite different environment from a single species of *Microcystis aeruginosa* used in the laboratory experiments. The morphological and physiological distinctions between colonies and isolated single cells should also be taken into account [35].

Among the MC-bound proteins identified by 2-DE immunoblots from semi-continuous culture samples (Table 1), phosphoribulokinase (PRK) is one of the essential enzymes of the Calvin cycle, and has also been identified as a MC-binding target in laboratory cultures of *Microcystis aeruginosa* PCC7806 [12]. PRK shows light/dark modulation of thiol oxidation and is regulated by both glutaredoxin and thioredoxin. Table S1 lists redox regulated proteins in *Synechocystis* sp. PCC6803 whose homologs were found to be MC-bound in our study [46,47,48,49]. Thioredoxin and glutaredoxin are the two main redox enzymes in cyanobacterial cells, modulating the activation status of many enzymes by the transient formation of disulfide bonds with their targets [47,48]. Furthermore, glutathionylation, through glutathione, also constitutes an important mechanism of redox signaling by protecting specific cysteine residues or by modulating protein activity [49]. MC bound to cysteine residues of RuBisCO could maintain its chemical conformation and activity under high light [12]. The protein-regulation characteristics of MCs may share some common features with those other redox regulators.

One important MC-bound protein identified was a 60 kDa chaperonin, named GroEL or RuBisCO-binding protein, belonging to the chaperonin (HSP60) family. GroEL is involved in the folding and assembly of multiple proteins, especially RuBisCO [50,51]. GroEL of *Microcystis aeruginosa* FACHB-905 has three cysteine residues, Cys 212, Cys 344 and Cys 519. Amino acid sequence alignment by ClustalX2 showed that Cys 519 is conserved in GroELs. The predicted structure of *Microcystis aeruginosa* FACHB-905 GroEL was obtained from SWISS-MODEL (model: PDB: 4V4O) (Figure S2). Cys519 is present at the surface of the protein, and can make contact with H_2_O and specific enzymes (Table S1). Microcystin may bind to Cys519 and maintain the biological activity of GroEL.

Zilliges et al. [12] identified RbcL as the main target of MC-binding in *Microcystis* *aeruginosa* PCC7806, which was not found in our studies. The spots on 2-D gels that were selected for characterization in the present study were those with highest signal intensity when detected by an anti-MC antibody. The signal of MC-bound RbcL may be weaker and therefore not selected for further analysis. Nevertheless, the binding of MC to PRK and GroEL, which are essential for RuBisCO activity, indicates that in semi-continuous culture of *Microcystis* FACHB-905, carbohydrate synthesis can also be regulated by MC. We identified several MC-bound proteins that were not identified in the report of Zilliges et al. [12], including fructose-bisphosphate aldolase (FBA), glyceraldehyde-3-phosphate dehydrogenase (GAPDH), phosphoglycerate kinase (PGK) and ATP synthase subunit alpha (AtpA), all of which participate in essential metabolic processes in cyanobacterial cells.

Glycolysis is a process of carbon catabolism that meets energy demands for the maintenance and growth of cells; the final product pyruvate, after decarboxylation to acetyl-CoA, takes part in several important cellular metabolic activities, including the tricarboxylic acid cycle, amino acid synthesis, and fatty acid metabolism. FBA, GAPDH and PGK catalyze three consecutive steps of the glycolytic pathway and are all essential enzymes; in addition to glycolysis, FBA and PGK also function in the Calvin cycle of CO_2_ assimilation in *Synechocystis* [52]. GAPDH has been found to be inactivated upon oxidation in various organisms including *Arabidopsis thaliana* [53]. PGK is nearly completely inactivated by oxidation, but can be easily reactivated by dithiothreitol (DTT) and thioredoxin [54]. These three essential glycolytic enzymes in *Synechocystis* are all regulated by thioredoxin and glutaredoxin, and can be modified by glutathionylation (Supplemental Table S1), which means their biological activity is sensitive to intracellular redox conditions. Another important MC-bound protein is AtpA, a component of ATP synthase. The ATP synthase complex is the primary cellular energy transduction and ATP synthesis enzyme in cyanobacterial cells. ATP synthase subunit alpha has been found to be subject to redox regulation in *Synechocystis* (Supplemental Table S1), and analysis of the level of thiol oxidation of AtpA showed that it was more reduced in light rather than dark conditions [46].

Thus, the MC-bound proteins we identified are mainly involved in carbon fixation, central metabolism and energy transduction. As mentioned above, an increasing quantity of MC bound to these proteins over time during steady-state growth in semi-continuous cultures. Together, these results imply that MC binding to these proteins may play an important role in sustaining growth in semi-continuous cultures. 

This preliminary investigation has detected a small portion of the protein pool to which MCs bind. A considerable number of MC-bound proteins and their functions are still unknown to us, and further in vitro investigations to identify these MC-bound proteins are needed. The discovery and understanding of the interaction of MCs with these proteins, as well as their biological roles, would assist in gaining further insight into the role of MCs in toxic cyanobacterial growth.

## 4. Materials and Methods

### 4.1. Cultures and Sampling

*Microcystis aeruginosa* FACHB-905 was obtained from the Freshwater Algae Culture Collection of the Institute of Hydrobiology (FACHB-collection, Wuhan, China). FACHB-905 was isolated from Lake Dianchi during the bloom period in 1998. The HPLC method was used to detect MC congeners of strain FACHB-905 and the result showed MC-LR was the only MC isoform, containing 3 mg per g of the weight of dry biomass. The cultures were maintained at 25 ± 1 °C in BG-11 medium [55] under a constant cool fluorescent light with an intensity of 25 µmol photons m^−2^·s^−1^, in a 12 h:12 h light:dark cycle.

Triplicate batch cultures (1.3 L cultures in 2-L Erlenmeyer flasks) were sampled (40–100 mL) every 3 days. Triplicate semi-continuous cultures (760 mL cultures in 1-L Erlenmeyer flasks) were sampled every 2 days, beginning on the eighth day after inoculation. For analysis of semi-continuous cultures, 25% of the culture was removed at the middle of the light period and was replaced with the same volume of fresh BG-11 medium.

Aliquots of 40–100 mL from batch cultures and from semi-continuous cultures were centrifuged twice at 3000× *g* for 6 min. The cell pellets from the two centrifugation runs were combined as intracellular cell components and the supernatants were combined as extracellular components. The combined pellet component, which contained intact *Microcystis* cells, was washed with sterile distilled water, and then immediately frozen in liquid nitrogen, lyophilized, and stored at −80 °C for later extraction of proteins and intracellular MCs. The extracellular component was filtered through a 47 mm glass microfiber membrane (1.2 μm, Whatman GF/C, GE Healthcare, Chicago, IL, USA) and the filtrate was immediately conducted for extracellular MCs concentrating.

Field samples were collected from a site at Meiliang Bay, Lake Taihu, near Shanghai, China (31.41° N, 120.19° E), one of the most eutrophicated bays in the lake. Samples were collected six times from 8 May 2013 to 5 December 2013, and four times from 28 July 2014 to 26 November 2014. Water samples were taken at a depth of 0.5 m with a small plankton net (64-μm mesh). The samples were rapidly frozen, freeze-dried, and stored at −80 °C for later MC extraction. Water temperature and pH of the sampling site were measured using an YSI Multiparameter Water Quality Sonde (6600 V2-4, YSI, Yellow Springs, OH, USA). Chlorophyll a was analyzed by standard methods [56].

### 4.2. Cell Counting

One mL samples of culture were fixed with 1 μL Lugol’s iodine solution. The fixed samples were mixed to homogeneity, placed in a hemacytometer (QiuJing factory, Shanghai, China), and counted under a microscope (Eclipse E200, Nikon, Tokyo, Japan). The counting was performed six times for each sample and cell concentration of each sample was calculated using the average cell number. The margin of error of the cell counts of each sample was <20%.

### 4.3. MC Analysis

Lyophylized powder from a known volume of culture was extracted once with 1 mL 5% acetic acid (*v*/*v*) for 40 min with continuous stirring. Additional extraction was performed twice with 1 mL 85% methanol (*v*/*v*) for 40 min. The pooled extract and the residue were each vacuum-dried. The dried residue of pooled extracts was then dissolved in 200 μL 50% methanol and kept at −20 °C in preparation for later HPLC analysis. Microcystin was extracted from field samples using the same protocol as for extracting laboratory cultures except that known weights of freeze-dried samples (approximately 10 mg) were ground to a powder under liquid N_2_ with a mortar and pestle prior to extraction.

The extracellular MC in the filtrate of extracellular component of batch and semi-continuous culture samples was concentrated and enriched by adsorption in a C18 Sep-Pak cartridge (Waters, Milford, MA, USA) and elution with 5 mL of HPLC-grade methanol. The eluent was evaporated to dryness with a mild flow of nitrogen gas, the residue was dissolved in 1 mL methanol, and the sample was again evaporated to dryness. The residue was then dissolved in 200 μL 50% methanol (*v*/*v*) and stored at −20 °C for future analysis by HPLC.

The MC content of samples was analyzed with an Alliance Waters™ e2695 (Waters, Milford, MA, USA) separation module coupled with a photodiode array (PDA) detector using a C18 cartridge (Prevail™ C18 Column, 250 × 4.6 mm, 5 μm, W. R. Grace and Company, Columbia, MD, USA). The column was maintained at 40 °C throughout the run. The mobile phase consisted of 40% phosphate buffer (50 mM KH_2_PO_4_, pH 3.0,) and 60% HPLC-grade methanol. The flow rate was 1 mL·min^−1^, and the constituents of the mobile phase were kept constant throughout the elution process. The injection volume was 10 μL. The MC content was monitored at 238 nm by recording UV spectra from 200 to 400 nm. Quantification was based on a standard curve established from linear regression values of authentic MC standards using Empower 3.0 (Waters, Milford, MA, USA). MC-LR and MC-RR standards were purchased from Wako Pure Chemical Industries (Osaka, Japan).

### 4.4. Sample Preparation and Immunoblot Analysis

Sample preparation was performed according to the protocol of Meissner et al. [22] with slight modification. The remaining debris after MC extraction, which contained the protein fraction, was dried with a mild flow of nitrogen gas to remove the remaining methanol. The residue was suspended in 500 μL sodium dodecyl sulfate (SDS) buffer (containing 0.5% SDS, 50 mM Tris-HCl pH 8.2, 10% glycerol), and then 1 M dithiothreitol (DTT) and 100 mM phenylmethanesulfonyl fluoride (PMSF) were added to a final concentrations of 20 mM and 1 mM, respectively. Proteins from the pellet fraction were solubilized by sonication for 3 min at 150 W, 20 kHz (VCX150, Sonics & Materials, Inc., Fairfield, CT, USA) in an ice-water bath, followed by centrifugation at 18,000× *g* for 40 min at 4 °C. The supernatant was transferred into fresh tubes, and the protein concentration was measured with Amido Black 10B [57] and bovine serum albumin as the standard.

Proteins were loaded onto 12% SDS-acrylamide gels [58], separated by electrophoresis and transferred onto polyvinylidene fluoride (PVDF) membranes (EMD Millipore, Billerica, MA, USA) at a constant current of 300 mA for 3 h in transfer buffer containing 20% (*v*/*v*) methanol. The membrane was blocked with 5% nonfat dry milk (Bio-Rad, Hercules, CA, USA) in TBS-T buffer (Tris-buffered saline pH 8.0 and 0.05% Tween^®^-20) at 4 °C overnight. Immunoblot analysis was performed with a specific anti-MC-LR antibody (prepared in our own laboratory, as described by Lei et al. [59] (incubated at a dilution of 1:200,000) for 2 h. The secondary horseradish peroxidase (HRP)-labeled antibody against mouse (Thermo Scientific, Rochester, NY, USA) was applied at a dilution of 1:2000 for 1 h. Detection of the signal was achieved by using Immobilon™ Western Chemiluminescent HRP Substrate (EMD Millipore, Billerica, MA, USA). Quantification was performed using Quantity One software (v4.4.0.36, Bio-Rad, Hercules, CA, USA).

### 4.5. Two-Dimensional Electrophoresis and Immunoblotting

Semi-continuous culture samples were used for two-dimensional electrophoresis (2-DE). The protein extraction procedures were in general the same as described above. However, the residues were suspended in lysis buffer specific for 2-DE (rehydration buffer (RB), consisting of 2 M thiourea, 7 M urea, 4% (*w/v*) 3-[(3-cholamidopropyl)dimethylammonio]-1-propane sulfonate (CHAPS), and additional PMSF to a final concentration of 1 mM) before use. Proteins in the supernatant were precipitated with four volumes of precooled acetone at −20 °C overnight and air-dried; the resulting protein powder was stored at −80 °C until further processing.

Before 2-DE, the protein samples were solubilized in 200 µL RB and the protein concentrations were measured by the Bradford method [60]. For analysis, two gels were run under the same conditions. The loading sample (including 500 μg protein, 1% DTT, 1% IPG buffer (pH 4–7, GE Healthcare, Chicago, IL, USA), and 0.1% bromophenol blue solution) was separated on Immobiline DryStrip Gels (pH 4–7, 180 mm, GE Healthcare) by first-dimension isoelectric focusing (IEF). The drystrip was rehydrated at 20 °C for 12 h before use and the IEF process was performed using a GE ETTAN IPGPHOR3 (GE Healthcare, Chicago, IL, USA)with a total of about 36 kVh. The second dimension separation was carried out on 12% SDS-PAGE (170 × 140 × 1 mm), using a GE ETTAN DALTsix (GE Healthcare). After 2-DE, gels were stained with Coomassie Brilliant Blue G250 and scanned (ScanMaker i800, MICROTEK, Shanghai, China) for further computational analysis. Separated proteins were transferred to a nitrocellulose blotting membrane (Pall Corporation, Port Washington, NY, USA) for immunological detection with anti-MC antibodies, as described above.

Gels and nitrocellulose blotting membranes were analyzed by ImageMaster 2D platinum 5.0 (GE Healthcare, Chicago, IL, USA). Spots with significant protein-bound MC signals were manually excised from Coomassie Blue-stained gel for further handling.

### 4.6. Mass Spectrometry (MS) Analysis and Protein Identification

Proteins in excised gel spots were digested with trypsin (Mass Spectrometry Grade, Promega, Madison, WI, USA) overnight at 37 °C. Peptides were extracted with 90% acetonitrile/2.5% trifluoroacetic acid and peptide masses were measured by matrix-assisted laser desorption/ionization time-of-flight/time-of-flight (MALDI-TOF/TOF) analysis (Ultraflex III MALDI-TOF/TOF, Bruker Daltonics, Billerica, MA, USA). The scanning UV wavelength was set at 355 nm and the mass scan range was 700 to 3200 Da.

MASCOT DAEMON (version 2.3.02, Matrix Science, London, UK) was used to extract the MS and MS/MS data via BioTools 3.0 software (Bruker Daltonics, Billerica, MA, USA). Database searches were performed in Mascot against the UniProt database (species: *Microcystis aeruginosa*, 79,051 sequences, 21,123,649 residues). The searches were performed with peptide mass tolerance of 50 ppm and fragment mass tolerance of 0.6 Da. One missing cleavage was allowed. Cysteine carbamidomethylation was set as a fixed modification, and methionine oxidation was set as a variable modification. Only significance thresholds defined by the Mascot probability analysis (*p* < 0.05) were accepted.

### 4.7. Statistical Analyses

All analyses were performed with one-way ANOVA (PASW Statistics 18, SPSS Inc., Chicago, IL, USA). For all results, the standard variance presented is ± one standard deviation (SD).

## Figures and Tables

**Table 1:** Dominant MC-bound proteins identified from semi-continuous culture samples.

Spot ^a^	Accession Number ^b^	Protein Name	Cys Residue ^c^	Mass (Mr) ^d^	pI Value ^e^	Score ^f^	Matched Peptides	Sequence Coverage	Functional Category
46	L8NVA2	Phosphoribulokinase	5	38,036	5.25	360	16	56%	Carbon metabolism
30	L8NKA9	Phosphoglycerate kinase	5	42,811	5.15	347	15	50%	Carbon metabolism
31	L8NQE4	Fructose-bisphosphate aldolase, class II, Calvin cycle subtype	4	39,156	5.4	368	15	45%	Carbon metabolism
32	L8NVZ5	Glyceraldehyde-3-phosphate dehydrogenase	5	37,128	5.76	190	8	29%	Carbon metabolism
8	L8NTF7	60 kDa chaperonin	3	57,701	5.15	414	21	50%	Protein folding and assembling
10	L8NV94	ATP synthase subunit alpha	3	54,116	5.02	533	25	46%	ATP biosynthesis
26	L8NP76	ThiF family protein	6	42,979	5.13	379	11	45%	Thiamin synthesis
70	L8NUD8	Acetyl-CoA acetyltransferases family protein	3	41,396	5.71	311	12	32%	Transferase, for acetylation

^a^ Spot number on the 2-DE gel; ^b^ Accession number from the UniProtKB database; ^c^ Cysteine residue number in the protein sequence; ^d^ Nominal mass; ^e^ Calculated pI value; ^f^ Protein score calculated by Mascot probability analysis.

## Supplementary Materials

The following are available online at www.mdpi.com/2072-6651/8/10/293/s1, Figure S1: Average monthly level of gross radiation intensity (□) and temperature (●) of months from January 2013 to December 2014. Data were provided by Taihu Laboratory for Lake Ecosystem Research, Chinese Ecosystem Research Network (meteorological station site: 31.42° N, 120.21° E). The meteorological station site is near our sampling site (31.41° N, 120.19° E) in Meiliang bay, Figure S2: Predicted structure of *Microcystis aeruginosa* FACHB-905 GroEL. The structure of *Microcystis aeruginosa* FACHB-905 GroEL was obtained using SWISS-MODEL. The model is based on the known structure of GroEL from *Thermus thermophilus* (PDB: 4V4O). The image of the three-dimensional structure was generated with SWISS-PdbViewer. The solute accessibility of three cysteine residues of *Microcystis aeruginosa* FACHB-905 in the image is indicated by the color as computed by SWISS-PdbViewer. Colors range from dark blue for completely buried amino acids to red for residues with maximum surface exposure, Table S1: Redox regulated proteins in *Synechocystis* sp. PCC6803.
