# Supplementary material for: Microcystin-Bound Protein Patterns in Different Cultures of Microcystis aeruginosa and Field Samples
Source: Toxins (Basel). 2016 Oct 12;8(10):293. doi: 10.3390/toxins8100293 (PMC5086653; doi:10.3390/toxins8100293)
Supplement: Supplementary file 1 [file toxins-08-00293-s001.pdf]

Supplementary Materials: Microcystin-Bound Protein Patterns in Different Cultures of *Microcystis aeruginosa* and Field Samples

Nian Wei, Lili Hu, Lirong Song and Nanqin Gan

Table S1. Redox regulated proteins in *Synechocystis* sp. PCC6803.

Protein Name	Accession Number [a]	ORF	Trx Target [b]	Grx Target [c]	Glutathionylated Regulation [d]	Redox Sensitive [e]
Phosphoribulokinase	P37101	sll1525	Y [47]	Y [48]	Y [49]	Y [46]
60 kDa chaperonin 1	Q05972	slr2076	Y [47]	Y [48]	Y [49]	-
Phosphoglycerate kinase	P74421	slr0394	Y [47]	Y [48]	Y [49]	Y [54]
Fructose-bisphosphate aldolase, class 2	Q55664	sll0018	Y [47]	-	Y [49]	Y [46]
ATP synthase subunit alpha	P27179	sll1326	Y [47]	Y [48]	Y [49]	Y [46]
Glyceraldehyde-3-phosphate dehydrogenase 1	P49433	slr0884	-	Y [48]	Y [49]	Y [46]

[a] Accession number in Uniprot database; [b] Protein identified as putative thioredoxin target in *Synechocystis* sp. PCC6803 [47]; [c] Protein identified as putative glutaredoxin target in *Synechocystis* sp. PCC6803 [48]; [d] Protein identified could be glutathionylated in *Synechocystis* sp. PCC6803 [49]; [e] Protein identified could be redox sensitive in *Synechocystis* sp. PCC6803 [46,54]. In the column, capital letter Y means the protein is under relative redox regulation in *Synechocystis* sp. PCC6803, and the reference is cited behind. The reference number is as listed as in the text.

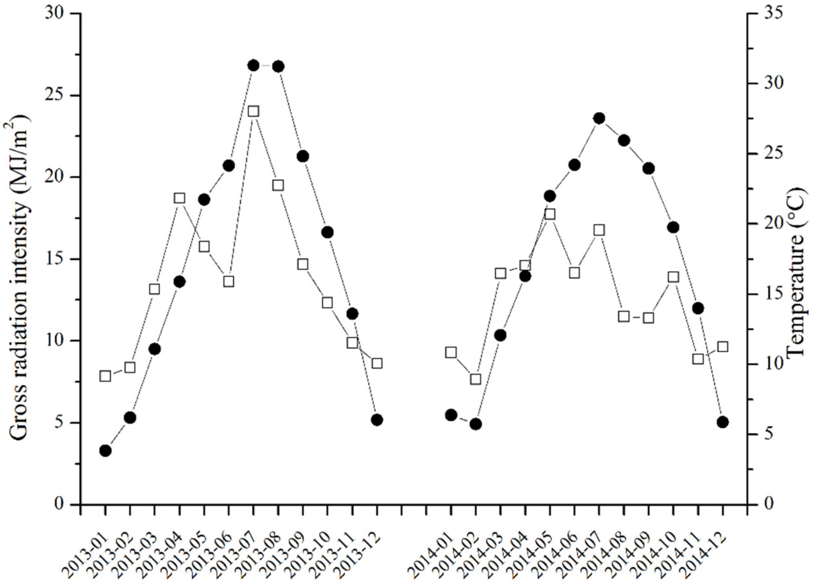

Figure S1. Average monthly level of gross radiation intensity (□) and temperature (●) of months from January 2013 to December 2014. Data were provided by Taihu Laboratory for Lake Ecosystem Research, Chinese Ecosystem Research Network (meteorological station site: 31.42° N, 120.21° E). The meteorological station site is near our sampling site (31.41° N, 120.19° E) in Meiliang bay.

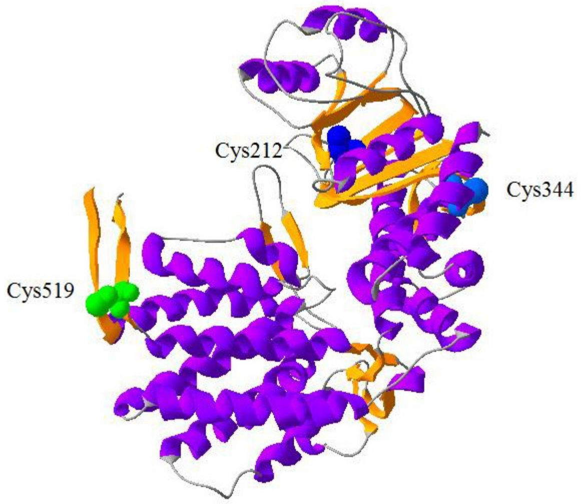

Figure S2. Predicted structure of *Microcystis aeruginosa* FACHB-905 GroEL. The structure of *Microcystis aeruginosa* FACHB-905 GroEL was obtained using SWISS-MODEL. The model is based on the known structure of GroEL from *Thermus thermophilus* (PDB: 4V4O). The image of the three-dimensional structure was generated with SWISS-PdbViewer. The solute accessibility of three cysteine residues of *Microcystis aeruginosa* FACHB-905 in the image is indicated by the color as computed by SWISS-PdbViewer. Colors range from dark blue for completely buried amino acids to red for residues with maximum surface exposure.
